# Arbuscular Mycorrhizal Fungi and Fertilization Influence Yield, Growth and Root Colonization of Different Tomato Genotype

**DOI:** 10.3390/plants11131743

**Published:** 2022-06-30

**Authors:** Zoltán Felföldi, Roxana Vidican, Vlad Stoian, Ioana A. Roman, Adriana F. Sestras, Teodor Rusu, Radu E. Sestras

**Affiliations:** 1 Department of Horticulture and Landscape, University of Agricultural Sciences and Veterinary Medicine of Cluj-Napoca, 3–5 Manastur St., 400372 Cluj-Napoca, Romania; zoltan.felfoldi@usamvcluj.ro (Z.F.); rsestras@usamvcluj.ro (R.E.S.); 2 Private Research Station Agrosel, 268 Laminoriștilor St., 400500 Câmpia Turzii, Romania; 3 Department of Microbiology, Faculty of Agriculture, University of Agricultural Sciences and Veterinary Medicine of Cluj-Napoca, 3–5 Manastur St., 400372 Cluj-Napoca, Romania; roxana.vidican@usamvcluj.ro (R.V.); vlad.stoian@usamvcluj.ro (V.S.); 4 Department of Transversal Competences, University of Agricultural Sciences and Veterinary Medicine Cluj-Napoca, 3–5 Manastur St., 400372 Cluj-Napoca, Romania; ioana.roman@usamvcluj.ro; 5 Department of Technical and Soil Sciences, Faculty of Agriculture, University of Agricultural Sciences and Veterinary Medicine of Cluj-Napoca, 3–5 Manastur St., 400372 Cluj-Napoca, Romania; trusu@usamvcluj.ro

**Keywords:** cultivar, mycorrhizae, nutrients uptake, soil analysis, soil microorganisms, tomato quality and yield

## Abstract

Arbuscular mycorrhizal fungi (AMF) are beneficial for plant development and help absorb water and minerals from the soil. The symbiosis between these fungi and plant roots is extremely important and could limit crop dependence on fertilizers. The aim of this study was to evaluate the influence of AMF on tomatoes (*Solanum lycopersicum* L.), based on important agronomic traits of vegetative biomass, production, and fruits. The experiment was conducted in high tunnels, using 12 tomato genotypes under three different treatments: T1, control, without fertilizer and mycorrhizae colonization; T2, fertigation, without mycorrhizae colonization; and T3, arbuscular mycorrhizal fungi (AMF), seedling roots being inoculated with specialized soil-borne fungi. Plant growth, yield and fruit parameters indicated better results under mycorrhizal treatment. Root colonization with fungi varied significantly depending on the treatment and genotype, with a variation of 6.0–80.3% for frequency and 2.6–24.6% for intensity. For a majority of characteristics, the mycorrhization (T3) induced significant differences compared with the T1 and T2 treatments. In addition, AMF treatment induced a different response among the genotypes. Among the elements analyzed in the soil, significant differences were observed in phosphorous levels between planting the seedlings and after tomato harvesting and clearing of the plants. The results suggest that reducing fertilizers and promoting the symbiotic relationships of plants with soil microorganisms may have beneficial consequences for tomato crops.

## 1. Introduction

Tomato (*Solanum lycopersicum* L.) is an important plant for human nutrition, and economically is one of the most important vegetables worldwide with approximately 182.3 million tons of tomato fruits produced on 4.85 million ha each year [1]. In intensive crops, the high production of tomatoes is also due to the large amounts of nitrogen and phosphate fertilizers that are applied [2]; however, phosphorus is a limited resource that can be depleted in about 40–70 years [3]. In recent years there has been great global interest in sustainable development of vegetable production systems that focus on high-quality vegetable crops and reduce the use of off-farm inputs, especially agrochemicals [4]. Crop production systems need to be modified to decrease the impact of crop production on the environment and for sustaining heightened levels of food production [5]. In the future, more sustainable cropping systems must be adopted to maintain high crop production and to protect and improve water and soil resources. These next model of cropping systems must focus on building soil health and minimizing the intake of synthetic fertilizers and pesticides [6,7]. A sustainable approach to agricultural practices includes the preservation of agricultural principles, such as minimizing soil destruction and the use of biodegradable foils. Adopting cover crops in cropping systems can provide several benefits, such as reducing the need for fertilizer, reducing the incidence of soilborne diseases by ensuring beneficial insect habitat, reducing herbicide use, and increasing crop yields by improving soil structure [8,9]. Numerous studies have shown that soil conservation works increase organic matter, soil structure and stability, which in turn reduce soil erosion and improve water retention and microbial and earthworm activity [10,11,12].

Currently, the biggest challenges facing agriculture are climate change [13], environmental pollution, accelerated degradation of ecosystems and the disappearance of soil biodiversity [14]. Agricultural production and productivity are closely linked and dependent on nutrient availability from the soil. For highly productive, quality crops, sometimes it is necessary to apply extra fertilizers. In fact, soil productivity and nutrient availability are interdependent. Basically, soil productivity is the ability of a soil to produce a certain yield of crops or other plants with a certain management system. The amounts of nutrients present in the soil are defined in terms of soil fertility; therefore, soil analysis is used as a criterion when making recommendations for extra fertilizers for open field crops [15]. Excessive application of chemical fertilizers can cause major imbalances that lead to ecological degradation in the underground environment [16]. In order to obtain high productivity and superior fruit quality, the soil quality is extremely important [17]. In tomatoes, because the soil quality can influence the entire crop throughout the year, the soil must be rich in nutrients and have an adequate depth and moisture for the plant to grow vigorously [18].

The rhizosphere is the habitat for a large community of microorganisms in the thin substrate of the soil near the roots [19], where most of the interactions between beneficial microorganisms and plant roots are found [20]. More than 80% of terrestrial plants show symbiosis with arbuscular mycorrhizal fungi (AMF) belonging to the phylum Glomeromycota. AMFs absorb photosynthetic byproducts and lipids through the plant symbiotic connections [21]. To improve crop production without further sacrificing environmental integrity, the use of AMF could contribute to enhancing yields in a more sustainable way [22,23]. These root symbionts provide plants with essential mineral nutrients, especially phosphorus (P), captured from the soil for their development in exchange for photo synthetics, sugars and lipids provided by plants [24].

The advantages gained by plants due to AMF interaction are not only an improved supply of nutrients, but also an increased resistance to soil pathogens and a high tolerance to stress against water, salt, and pollutants [25,26,27]. Other beneficial effects are early flowering and an increase in marketable fruits [28,29,30,31,32,33]. As a result, AMF is increasingly integrated into plant production systems, as it helps reduce the application of chemical fertilizers and pesticides and is, therefore, an important component of sustainable food production [34]. Thus, these microbes that contain AMF as “bio stimulants” could provide a safe solution to meet the future challenges in human nutrition mentioned above [35]. Host plant susceptibility to AMF is highly variable and depends on many factors, including plant genotype. Consequently, tomato breeding should be pursued by selecting varieties showing high mycorrhizal susceptibility [36,37,38].

Therefore, our study aimed to determine the possible growing conditions of tomatoes to reduce the application of chemical fertilizers in favor of the use of arbuscular mycorrhiza fungi. We investigated how AMF can ensure plant development, nutrient uptake, and production in several tomato genotypes, including two newly created commercial hybrids. In order to achieve the proposed objectives and to understand mycorrhizal colonization, we investigated the effects of AMF in three high tunnels located very close to each other, in which three different treatments were applied.

## 2. Results

### 2.1. Soil Sample Results and Differences of Soil Components before and after Cultures

The main components in the soil after culture and clearing of plants at the end of the crop were different from those at the start of planting, the differences oscillating depending on the treatments (Table 1).

Considering the mean values of the three treatments (T1, T2 and T3) for the twenty-seven soil components analyzed before and after culture (Table 2), the paired *t*-test showed positive changes in soil composition due to tomato cultivation (soil interaction with plants) for phosphorus content. For the other soil components, there were no significant changes in the content at the end of cultivation. Accordingly, when the plantation was cleared and plant material and plant debris were removed, the soil indicated the same content values as at the beginning of cultivation.

### 2.2. Parameters of Plant Growth, Fruit Production and Quality

The results for biomass accumulation represented by root length, plant height and the number of leaves per plant indicated significant differences among genotype × treatment combinations, but also among genotypes and treatments (Table 3).

Among the interactions genotypes × treatment, for root length, the highest values were reported for line AS30♀F7 in the T3 treatment (29.2 cm), followed by line AS09♀F7 also in T3, with a very similar value (28.9 cm). The highest value for plant height was found in T3 in the line AS09♀F7 (149.0 cm), and the highest number of leaves per plant was recorded for the commercial hybrids AS400 (13), also in T3. The unilateral analysis of the effect of the genotype on the analyzed characteristics, regardless of the treatment, highlights a significant influence of the hereditary dowry of the cultivar on the elements that contribute to the growth of tomatoes. The highest average values for root length were recorded for AS30♀F7, the highest plants for AS 400, and the most leaves per plant for AS300. The unilateral effect of the treatments significantly influenced all three elements of plant biomass. The differences between the average values of the three growth elements were significant, the hierarchy of treatments being the same: T3 > T2 > T1.

Dry root weight and dry shoots weight showed significant differences among the genotype × treatment interactions (values with lowercase letters), genotypes (in the column with the averages resulting from the three treatments, with capital letters), and treatments (on the bottom row, with the averages resulting from all the genotypes, where the differences are marked with capital letters) (Table 4). The effects of the treatments were obvious. The plants that were inoculated with mycorrhiza have predominantly higher values. The resulting order for the influence of the treatments on the two traits was the same as for the previous traits: T3 > T2 > T1.

The two experimental factors (genotype and treatment) and their interaction significantly influenced the main characteristics of the fruits, according to the results in Table 5. The unilateral effect of the treatment was extremely different from one character to another with the following order of treatment effects: T1 > T2 > T3 (fruit height); T1 > T3 > T2 (fruit width); T1 > T2 > T3 (fruit shape index); and T3 > T2 > T1 (fruit weight). Consequently, the T3 treatment (arbuscular mycorrhizal fungi) produced tomatoes with the lowest height and the lowest shape index, but with the highest weight.

The number of fruits per plant and fruit production (per plant and per area) fluctuated strongly. Clearly, both the genotype, treatment and their interaction significantly influenced the results (Table 6). The number of fruits per plant as the mean of T1, T2 and T3 treatments varied significantly between genotypes from 10.0 to 18.8, production per plant from 1.3 to 4.2 kg, and production per area (with a density of 3.3 plants/1 m^2^) from 4.2 to 13.8 kg. The highest values of yield per plant were recorded for the new commercial hybrid AS400 under the T2 treatment (4.35 kg/plant), and under the T3 treatment (4.15 kg/plant). The unilateral effect of the treatment was significant on the loading of the plants with fruits, the lack of fertilization and mycorrhization (AMF) determining the highest number of fruits per plant, with the following order of treatments: T1 > T2 > T3. Fruit production, both per plant and per area, was superior in mycorrhizal and fertilization conditions as follows: T3 > T2 > T1.

The interaction between genotype and treatment influenced the two elements of fruit quality, with variation between 4.0 and 7.0 for fruit firmness, and 4.5 and 5.4 for fruit sugar content (Table 7). Both the genotype and the treatment significantly influenced the firmness of the tomatoes and their sugar content. The AMF treatment (T3) had a favorable effect on the two characteristics, whereas the fertilization treatment (T2) decreased the values of the two elements of fruit quality according to the following order: T3 > T1 > T2.

### 2.3. Colonization Parameters As a Genotype Reaction to Mycorrhizal Inoculation and Treatment Influence

Mycorrhizal colonization varied widely depending on both the treatment and genotype, which formed a dynamic interaction (Table 8). The colonization frequency varied widely in the range 6.0–80.3%, with generally higher values when mycorrhizal treatment was applied. The absence of treatments (controls), permits the observation of the native mycorrhizal acceptance potential of the studied genotypes. This value exceeds 50% in the case of AS30♀F7 and Addalyn genotypes, with other four genotypes in the range 40–50%. The lowest values of this parameter, under 20% presence of fungi in roots, were recorded only in two cases: AS28♀F6 and AS400. The application of fertilizers induces a reduction of colonization frequency with AS07♀F6 showing values five time lower than the control and the mycorrhized treatment. The general trend observed in the experiment shows a 2- to 3-fold decrease in colonization frequency compared with the native potential of genotypes.

The application of mycorrhizal inoculum acts in two directions: it either induces an increase in the frequency compared with the native potential or decreases the frequency, in some cases to levels similar to those for fertilizers. From the genotypes that have a higher potential to use fungal inoculum, AS30♀F7 had an increase of 26% compared with the control and an almost 36% increase compared with the fertilized treatment. In contrast, the AS09♀F7 and Addalyn genotypes had drastically reduced frequencies (more than three-fold reductions compared with the control). The most interesting case is represented by the genotype AS300, a genotype where the fertilizers induced an increase of colonization frequency with more than 5% compared with the control, but this genotype showed an extremely reduced permissiveness to the mycorrhizal inoculum at 6%.

The variation of colonization frequency permits the separation of genotypes into four classes: high native acceptance potential, high acceptance of inoculum, acceptance rate increased by fertilizers, and low acceptance rate of inoculum. The trends in intensity of colonization showed values 3–5 times lower than their corresponding frequencies. Only some exceptions presented higher intensities, including AS28♀F6 with an intensity only 2% lower than the frequency. In the case of the fertilized treatment and commercial hybrid AS400, the difference between frequency and intensity was only 6%, suggesting a low acceptance of mycorrhizal inoculum and better use of fertilizers as a stimulus for fungal colonization.

Due to the low acceptance of fungal partners, the non-mycorrhizal areas occupied more than 80–90%, with only one case (AS30♀F7) at 75%. This phenomenon represents a large area of roots where the fungal development is blocked and the symbiosis fails to install. For the same AS30♀F7 genotype, the colonization degree reached almost 20%, a significantly higher value compared with the rest of the treatment. This method is the most suitable for the application of the inoculum and results in a more than 10% increase in the degree of colonization compared with other methods.

**Figure 1:**
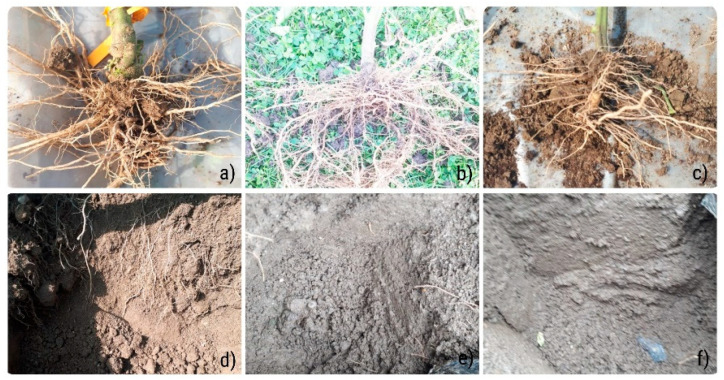
Differences induced by treatments in fresh tomato plant roots: (**a**) roots treated with mycorrhiza; (**b**) fertilized roots; (**c**) control roots (with no treatment). Soil profile containing thin roots after the extraction of plant; (**d**) soil of mycorrhizal treatment; (**e**) fertilized soil and (**f**) control soil (without treatments).

After completing the vegetative cycle and after the plants were extracted from the soil in order to analyze the roots, differences were observed in the soil wall under the three different treatments. As shown in Figure 1, the first three pictures above (Figure 1a–c) presented the roots extracted from the soil from the three treatments, respectively, and the pictures below (Figure 1d–f) were the locations from which they were extracted. It was considered and validated that the early administration of these beneficial fungi leads to an intensified development of plant roots and helps plants to assimilate nutrients from the soil.

**Figure 2:**
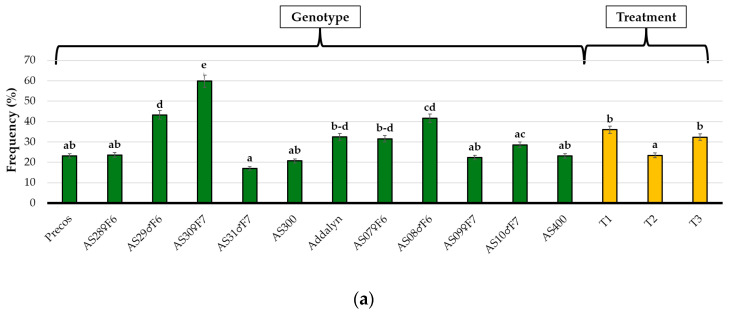

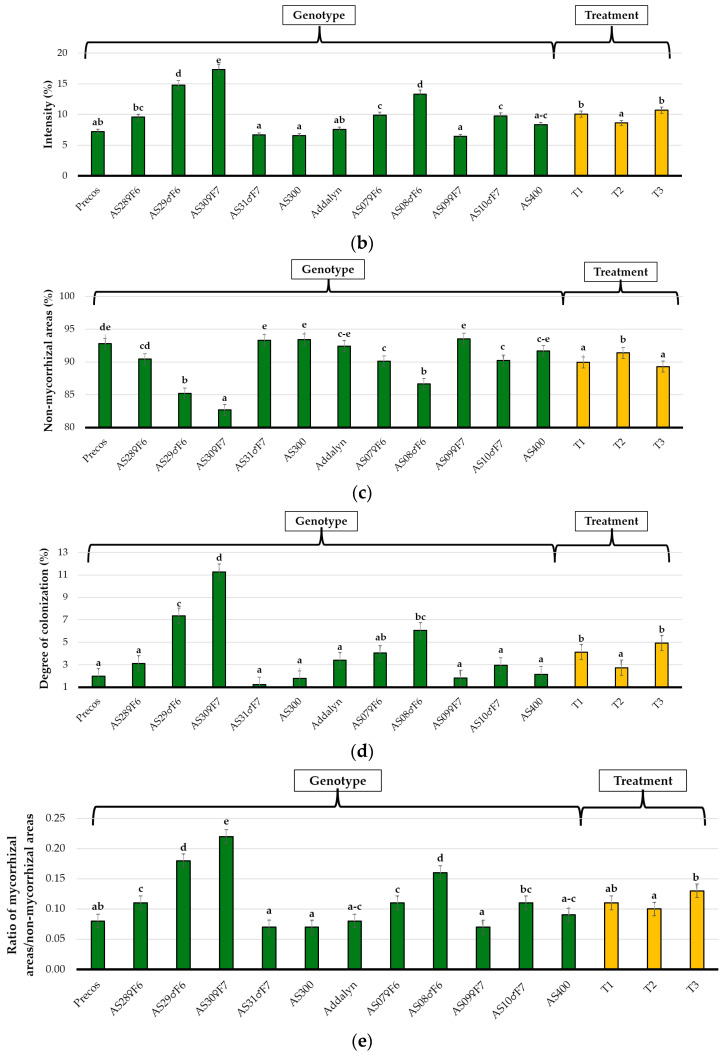
Tomato root colonization analysis under the influence of genotype and treatment: (**a**) average trend of colonization frequency; (**b**) average trend of colonization intensity; (**c**) average trend of non-mycorrhizal area; (**d**) average trend of colonization degree; (**e**) average trend of mycorrhizal/non-mycorrhizal areas. Explanations for the three treatments (T1, T2, and T3) are presented in the caption of Table 1. Genotype and treatment values are indicated by green and yellow, respectively. Significant differences between means are illustrated with different letters (Duncan’s Multiple Range Test, α < 0.05).

The average trend of colonization frequency varied greatly between genotypes and between treatments (Figure 2a). The most performant genotype was AS30♀F7, with an average frequency of over 60%. This was significantly higher compared with the second genotype AS29♂F6, which had a 20% lower value. A group of three genotypes (Addalyn, AS07♀F6 and AS♂08F6) had values in the range 30–40%, but the differences compared with the rest of the genotypes were not significant. The only exception was AS31♂F7 with an average frequency lower than 20%, which was the minimum average for the entire set of genotypes. Of the three treatments, two did not show significant differences (T1 and T3), but both were significantly higher compared with T2. An interesting aspect was that the genotype set has a native mycorrhizal acceptance of over 35%, which was over 5% higher, but not significant, than the acceptance of the inoculum. Both these rates are significantly higher than the mycorrhizal acceptance of genotypes to which fertilizer was applied. This phenomenon is related to the reduction in the permissiveness of roots for fungal colonization due to the increased availability of nutrients in the root area.

The maximum colonization intensity depending on genotype (Figure 2b) was 17% for AS30♀F7, which was significantly higher than for AS29♂F6 and AS08♂F6 with values of this parameter in the range 10–15%. The rest of the genotypes had the intensity values in the range 5–10%, three of them with values in the upper part of this range (AS28♀F6, AS07♀F6 and AS10♂F7). The intensity recorded for treatments followed the same trend as for the frequency with no differences between T1 and T3, and both being significantly superior compared with T2. Inoculation of plants slightly increased the intensity value of colonization compared with the native potential. Both reached around 10%, whereas the application of fertilizers set this value at 8%, which was significantly lower compared with the control and inoculated treatments.

The application of fertilizers raises the presence of non-mycorrhizal areas to almost 92%, a value significantly higher than the native presence of these areas and compared with the application of inoculum (Figure 2c). Four genotypes (Precos, AS31♂F7, AS300 and AS09♀F7) had values of this parameter in the range 92–94%, which makes them less suitable for the establishment of a functional symbiosis. Compared with these four genotypes, AS28♀F6, AS07♀F6 and AS10♂F7 showed decreasing values of non-mycorrhizal areas, a lowering trend that maintained this parameter at 90%. Once this threshold was exceeded, for the AS08♂F6, AS29♂F6 and AS30♀F7 genotypes, a decreasing trend in non-mycorrhizal areas of up to 82% was observed.

A 10% range (1–11%) in colonization degree was observed (Figure 2d) due to the specific genotype reaction. The hierarchy of genotypes shows a maximum of 11% reached by AS30♀F7, a significant decrease of 4% in AS29♂F6 genotypes, followed by an insignificant reduction in two genotypes: AS08♂F7 and AS07♀F6. Below 3% of colonization degree, the differences recorded between genotypes lack significance. The inoculum’s influence was measure to be 5% colonization degree for the entire set of genotypes, which was 1% higher than the native potential and almost 2% significantly higher than the fertilized ones.

In the assessment of the genotype reaction to applied treatments, a significant variation in mycorrhizal/non-mycorrhizal areas was observed (Figure 2e). The highest value was observed in genotype AS30♂F7, the only genotype with values over 0.2, and which was significantly higher than AS29♂F6 and AS08♂F6, both with values near to 0.2. All the other tested genotypes showed values of almost 0.1 and lower, but with differences considered not significant. Overall, the treatment effect was significantly higher for the inoculated genotypes com-pared with fertilized ones, and with the native potential having intermediate values.

### 2.4. Correlations between the Analyzed Characteristics and the Multivariate Analysis

**Figure 3:**
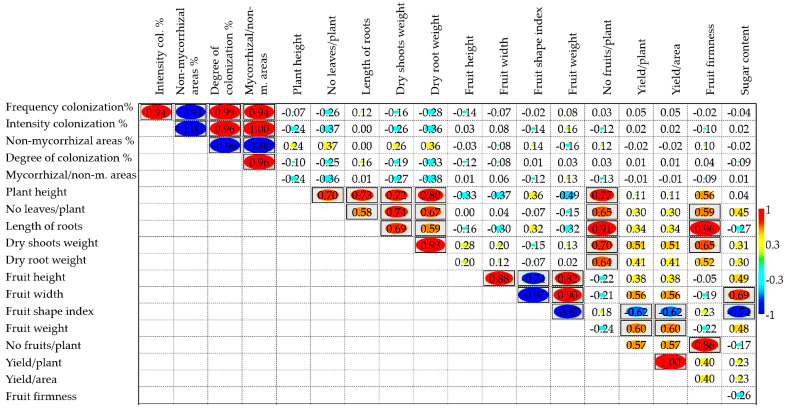
Phenotypic correlations between the pairs of traits analyzed. Correlations were calculated from mean values of the traits. Positive correlations are displayed in blue and negative correlations in red. Color intensity and the size of the circle are proportional to the correlation coefficients. The grey background boxes illustrate the significant values at the level of *p* < 0.05 (2-tailed).

Pearson correlation coefficients allowed the identification of close, statistically significant relationships (*p* < 0.05) between all mycorrhizal parameters (Figure 3). The frequency of colonization was positively and strongly correlated with the intensity and degree of colonization, as well as with mycorrhizal/non-mycorrhizal areas, and the intensity of colonization with the degree of colonization and with mycorrhizal/non-mycorrhizal areas. The elements that contribute to the accumulation of biomass have been positively correlated with each other, these having a significant correlation with the number of fruits per plant. Three of them (number of leaves per plant, length of roots and dry shoots weight) were also significantly correlated with fruit firmness.

In addition to the anticipated correlations between fruit shape index and morphological elements of fruits (e.g., fruit height and fruit width), significant but less predictable negative correlations were identified between this index and fruit weight, yield per plant, yield per area, and the sugar content of the fruit. Other directly proportional correlations suggests that, in this study, larger diameter fruits had higher sugar Brix values, and fruit weight was correlated with yield per plant and yield per area. Finally, the firmness of the fruit was positively correlated with the length of the roots, the dry shoots weight, and the number of leaves per plant, and these correlations were less expected.

**Figure 4:**
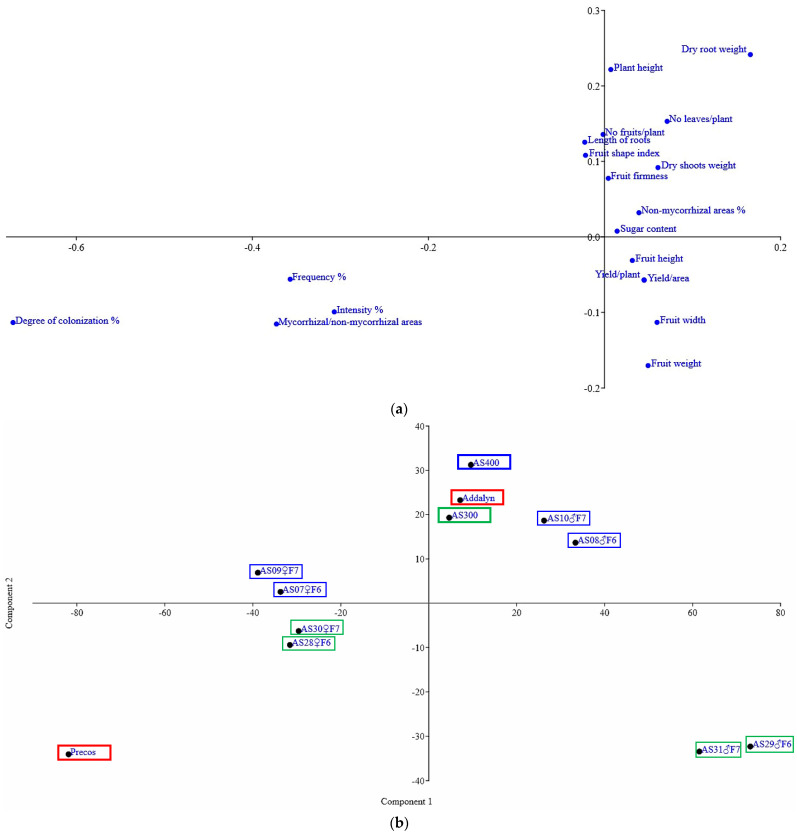

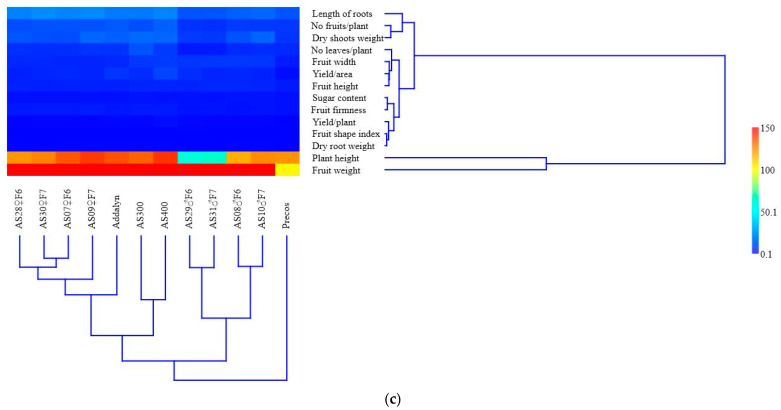
Multivariate analyses performed for traits of plant growth, yield, and fruit quality in 12 tomato genotypes: (**a**) Correspondence analysis (CA), 19 parameters analyzed; (**b**) Principal Component Analysis (PCA), 19 parameters analyzed; (**c**) Hierarchical clustering, paired group UPGMA (Unweighted Pair Group Method with Arithmetic Mean), similarity index (Gower), 14 parameters analyzed (without mycorrhizal ones).

The results obtained by correspondence analysis (CA) are presented in Figure 4a. The two-dimensional CA provides an impactful display of all data and an overview of how mycorrhizal parameters are grouped close together, in the lower-left part of the graph. Mycorrhizal parameters are in opposition to most elements of plant growth and fruit quality, while fruit characters (height, width, and diameter), as well as production, are grouped separately, quite closely, in the lower right of the graph. In the CA dataset, axis 1 explains 55.8% and axis 2, 32.3% of the variation, so cumulatively, the two axes contribute 88.1% of the total variation.

Principal component analysis (PCA) performed for the 19 analyzed parameters (Figure 4b) offered a particularly interesting arrangement of genotypes. The newly obtained F1 commercial hybrids (AS 300, framed in a green rectangle, and AS400 in a blue rectangle) were grouped quite closely together, along with one of the hybrids used as a control in their creation process (Addalyn, red rectangle). In contrast, all of them were in the opposite position to Precos (red rectangle), the second commercial hybrid used as a control. Precos was positioned in an obvious negative correlation, in a quadrant on an opposite diagonal to the other commercial hybrids.

The parental lines of the two new commercial hybrids (in rectangles that respect the filial color) are spread in all four quadrants of the PCA, but in pairs and arranged above or below the horizontal axis which confirms the genetic approximations according to the breeding scheme presented in our previous research [39]. Thus, the parental lines closely grouped in pairs, confirm that the phenotype represented by the 19 parameters analyzed accurately reflects their genotype. Each pair is represented by the same line, but in generations F6 and F7 when they had a high degree of homozygosity following the selection process, so that the characteristics based on which they were grouped so closely were extremely similar, or identical. The heterosis effect due to the pronounced heterozygosity of commercial hybrids is very well reflected by the PCA, especially in the F1 hybrid AS300. The parental forms are in different quadrants, both under the horizontal axis. In addition, the maternal line (AS28♀F6 and AS30♀F7, the latter entering the parental formula, together with the paternal line, AS31♂F7) is in the opposite quadrant, illustrating an inversely proportional relationship. In the PCA, the first axis explains about 73.3% of the data, and the second axis explains 19.5%.

## 3. Discussion

The investigations of the literature related to our study illustrate that symbiosis between tomatoes and beneficial fungi, also called mycorrhizae, are essential in the development of tomato plants [40,41]. A similar feature of these symbioses is the evidence of mutual recognition between terrestrial plants and bacterial or fungal microorganisms prior to host-regulated microbial entry [42]. Thus, these symbionts modify the resistance of plants to disease, reduce the impact of pathogens and improve the overall integrity of crops [43,44]. They also act as indirect support for plant growth and development [45,46], by changing the phosphorus mechanism and partially the replacing use of fertilizers.

The beneficial effect of arbuscular mycorrhiza on tomato plants [47,48,49,50] is often linked to higher efficiency of mycorrhizal plants in the absorption of nutrients from the soil, especially phosphorus. In our study, the contribution of mycorrhizae to phosphorus uptake in soil [24] has been confirmed. Statistically, the phosphorus content of the soil after the completion of the tomato crop was higher than the existing soil content at the beginning of the crop. Because P uptake in plants occurs primarily during early-season growth [51], AMF may enhance early-season P acquisition [52]. We also consider that, in addition to the five beneficial fungi, the other additional biopreparations of the product that we used in our study contributed to the improvement of nutrient absorption, having a synergistic action.

Our results indicate various changes in plant growth parameters depending on the fertilizers and AMF, the development of mycorrhizae in the root system and a large variation in the colonization parameters in the area where the tomato seedlings were inoculated with mycorrhiza. The results obtained in the three treatments indicated significant improvements in the total root length, dry root weight and dry shoot weight of the root, when AMF was used, compared with the controls (untreated, no fertilizers and AMF). These results are comparable with the results obtained by Berta et al. [53], who also concluded that inoculation with AMF has an essential role in the development of roots. Improvement of the vegetative growth of tomato plants may be attributed to the growing conditions and the good condition of the plants, which facilitates the uptake of nutrients, and improves the photosynthesis and transport of metabolites [54].

The yield increase promoted by AMF inoculation is connected to the biostimulant action of these fungi on plant uptake and growth [55,56]. In this respect, plant growth and yield are dependent on the nutrient availability during the phenological development [57,58]. In our study, we found a significant increase in mycorrhizal plants in terms of yield in the twelve tomato genotypes. The result confirms previous studies, which showed a clear increase in the yield of mycorrhizal tomato plants compared with non-mycorrhizal plants [28,59,60]. Considering the production after inoculation of plants, the results indicated similar values to those observed under the influence of the fertilizer treatment; similar results have been observed in other studies [61,62,63,64,65,66,67,68,69,70,71]. It was also found that the quantity and quality of pollen in the flowers are closely related to the mycorrhizal colonization of plants [43]. This may be an additional reason that plants inoculated with mycorrhiza improve the growth, development, yield and quality of tomatoes [72,73,74].

In the context of new agronomic adaptative strategies [75,76], which emerged in the last decades, the variation in colonization parameters should be used as a separation of genotypes in multiple classes. In previous studies, mycorrhizal inoculation significantly increased the levels of colonization of tomato roots compared with uninoculated plants [69]. The growth improvement is also probably due to pre-inoculation [77]. The increase in colonization will sustain a better resistance to biotic and abiotic stresses [62,78,79,80]. However, there are genotypes with low inoculum acceptance rates that correlate with low mycorrhizal potential. Consequently, new research should be conducted on these genotypes, in order to explore new sources of inoculum, to identify other microbial auxiliary sources (e.g., bacteria), to ensure mycorrhizal success [81,82], or to identify new fertilization strategies that may alleviate the absence of fungal symbionts at the root system.

The frequency of colonization in our study was lower than that reported by Makaboko et al. [83], who observed a colonization frequency of 78.2% for roots of tomato plants grown in coconut and 77.7% in sawdust. In contrast, Cwala et al. [84], obtained a lower colonization frequency, between 14 to 25%, but in hydroponic culture. The intensity of colonization was influenced by the genotypes we used, with a variation of between 10 and 15%. The trend for colonization intensity was similar to that for frequency, without differences between T1 and T3, but both were significantly higher than for T2. A low level of arbuscular intensity may suggest that there is no excess of substances between the plant and the fungus, which can reduce the benefits of mycorrhizae, including improving the nutritional status of plants [85]. The degree of colonization of the roots by endomycorrhizal fungi has an influence on the quantities of nutrients provided to the plant. This aspect causes changes in the quality and quantity of root exudates produced by mycorrhizal plants [78,86]. The degree of colonization in our experiment ranged from 1 to 11%. The inoculum was observed to have a colonization rate of 5% for the whole set of genotypes, 1% higher than the native potential and almost 2% significantly higher than in the fertilized treatments. In other experiments performed by Kowalska et al. [85], the relative abundance of the degree of colonization was at a very low level, with values between 0.02 and 0.07% in non-inoculated plants, and a range of 1.01–2.51% in plants subjected to inoculation.

The response of plants to AMF treatment in our study provides original data, due to the genetic material and multivariate analysis used to generate the results. Although mycorrhization resulted in relatively large standard deviations due to the three types of treatments performed, multivariate analysis (PCA) grouped the parental lines of the two new commercial hybrids very closely. The component lines of the two new commercial hybrids in the advanced F6 and F7 generations have a homogeneous genetic structure, with a high degree of homozygosity. Their genetic uniformity was faithfully confirmed in the PCA, which summarized proportions of the variation of the analyzed parameters including their reaction to mycorrhization, which represented five of the nineteen parameters analyzed. Therefore, the tight grouping in the PCA graph of these homozygous lines, by grouping in F6-F7 pairs, reflects the clear and reliable differentiation of genotypes to AMF. This is also strong evidence that a particular reaction of tomato cultivars to AMF can be identified with sufficient precision. The result also reflects the reliability of the mycorrhizal evaluation model used in the present experiment.

In our research, it was observed that there was an improvement in the quality of tomato fruits and a higher percentage of production following the application of arbuscular mycorrhizal fungi (AMF), which are beneficial for biodiversity and the ecosystem. Given the high expectations of consumers for healthy products as well as current policies aimed at organic farming systems, the use of these beneficial microorganisms is an efficient and environmentally friendly farming technique that aims to reduce the application of chemical fertilizers. It is possible to enhance the results obtained by using AMF by selection of certain genotypes. Our research has shown a significant effect of genotype on the plant vigor, fruit production and some quality elements of tomatoes. Certainly, the identification and promotion of the cultivation of such genotypes under the conditions of mycorrhizal inoculation can contribute to increasing the efficiency of tomato cultivation and its sustainability.

## 4. Materials and Methods

### 4.1. Microclimatological Measurements Using Climate Stations

**Figure 5:**
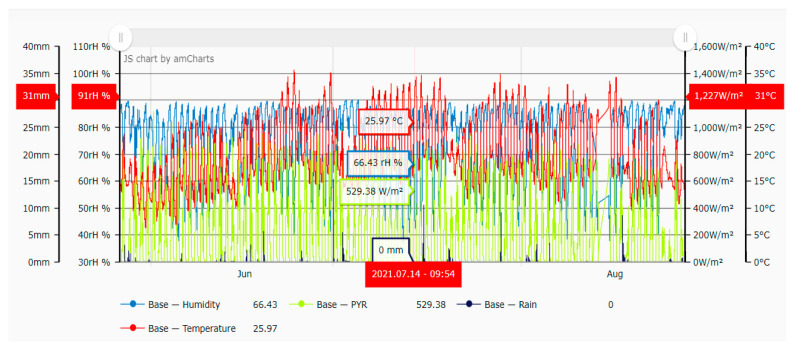
Microclimatological measurements from the experimental period for temperature, humidity, solar radiation, and rain values.

Two weather stations were installed: the Agrosense Base and Agrosense Node + Air (Sys–Control Informatikai Kft., Hungary). Connected sensor data were transmitted to the Ag-rosense Base agricultural meteorological station by radio signals. This climate station was used to supplement the Agrosense Base meteorology and microclimatic parameters to accurately measure the most important parameters for plant development in the immediate vicinity of the plants. The micrometeorological variables collected by the agricultural meteorological station during the experimental period indicated differences related to air temperature (oscillating between 6.5 and 38.5 °C) and relative humidity (between 37.5 and 90.3 rH%) in the high tunnels of three treatments. There were some differences in the average solar radiation between tunnels but these were extremely small. Rainfall values were calculated in relation to the unit area/per square meter (1 mm of water measured is equal to 1 Liter of rain-water) and ranged from 0 to 20 mm. Micrometeorological variable data were collected at 30 min intervals from 15 May to 30 August (Figure 5, exemplification for high tunnel no. 1, T1 treatment).

### 4.2. Field Location and Experimental Design

The experiments were carried out using a polyfactorial design under three different treatments: T1, control, without fertilizer and mycorrhizae colonization; T2, NPK and microelements fertigation without mycorrhizae colonization; and T3, arbuscular mycorrhizal fungi (AMF), seedling roots inoculated with specialized soil-borne fungi. The plants were arranged in a randomized complete block design with three repetitions.

The experiment was performed at the Agrosel Research Station, in Campia Turzii, in Cluj County, north-western Romania (46°32′55″ lat. N and 23°52′48″ long E). The tomato genotypes were grown in high tunnels covered with insect net and oriented in a north–south direction for the summer–autumn season under the same conditions, but under different fertilization. The tunnels had an area of 128 m², equipped with black biodegradable mulch foil. An automatic fertigation system ITU Mix Station 300 (Itumic Oy, Finland) was used for fertilizer administration.

At 39 days before transplanting, the tomato seedlings were inoculated with AMF (T3 treatment). The inoculation was carried out by arbuscular mycorrhizal fungi corresponding to a commercial product Symbivit (Symbiom Ltd., Lanskroun, Czech Republic), which contains a mixture of spores and mycelium of *Rhizophagus irregularis*, *Claroideoglomus etunicatum*, *Claroideoglomus claroideum*, *Funneliformis mosseae* and *Funneliformis geosporum*. Except for the bioactive particles represented by these five mycorrhizal fungi (which are living in European soils), the product contained bioaditive particles (18.4%), including keratin, ground phosphates, sapropel, alginates (seaweed), gumat, dolomite, urea, and water storage granules.

The tomato seedlings were transplanted into the field on 10 May 2021 in twin rows, with 0.35 m spacing inside the rows, 0.70 m between adjacent twin rows and 0.90 m between the rows. During the transplantation into the field, the seedlings of three repetitions of 12 tomato genotypes in T3 were inoculated by adding 20 g of Symbivit product per plant into the planting filed, near the roots. For the preparation of this composition, 4 kg of Klasmann TS3 extra fine peat, 4.32 kg of Symbivit mycorrhiza and a volume of 15 litter of water were used. A uniform mix was made of all these three components.

Post harvesting, 36 plants from each tunnel were randomly uprooted from the ground to determine the parameters of the root system. The weight of the roots, length of roots, frequency of mycorrhizae in the root system (%), intensity of mycorrhizal colonization in the root system (%), intensity of mycorrhizal colonization in root fragments (%), non-mycorrhizal areas (%), degree of colonization (%) and ratio of mycorrhizal areas/non-mycorrhizal areas were measured.

### 4.3. Biological Material

The plant material was represented by twelve tomato genotypes, with medium to large fruits, of which four were F1 commercial hybrids and eight lines in advanced generations of selection in the breeding process. Of the four commercial hybrids, two were obtained recently at the Agrosel Research Station (AS300 and AS400), and two were commercial hybrids, were are widely cultivated in Romania and were used in the experiment as controls (F1 hybrids Precos and Addalyn, produced by Geosem and Hazera Seeds, respectively). The eight lines included in the experiment were the parental lines of the commercial hybrids AS300 and AS400 in F7 and F6 generations, respectively. The female and male lines are represented with parental symbols ♀ and ♂ [39].

### 4.4. Fertigation Plan and Nutrition Data

Fertilization throughout the vegetation period was performed with the automated fertilization system ITU Mix Station 300 (Itumic Oy, Finland). The fertilizers (T2 treatment) were administered in different doses at three different stages of vegetation (after planting, in flowering stages, and in ripening stages). The complex fertilizers were administered in different dosages in three stages of vegetation (from planting to flowering at 1.5 g/plant/day; from flowering to fruit harvesting at 3 g/plant/day; from fruit harvesting until the last harvest at 4 g/plant/day). The quantities of fertilizers expressed in kg were mixed in separate tanks: A, tanks with nitrates; and B, tanks with sulphates. The tanks had a capacity of 500 L. The total amount of the fertilizers applied throughout the experimental period was: Ca(NO_3_)_2_: 6 kg; NH_4_NO_3_: 4 kg; KNO_3_: 8 kg; CaO, MgO, B, Cu, Fe, Mn, Mo, Zn: 3 kg; KH_2_PO_4_: 7 kg; K_2_SO_4_: 6 kg; and MgSO_4_: 5 kg.

### 4.5. Laboratory and Microscopic Analysis

After the plants had been extracted from the soil, the roots were brought to the laboratory and prepared for the determination of mycorrhizal colonization parameters. For the microscopic analysis of tomato roots, the staining method described by [87] was used, in which agents such as ink, vinegar, and caustic soda are used to observe the intra radicular structures of mycorrhizal fungi. The four steps of the method are cleaning, rinsing, staining and destaining [88], with the use of boiled water to dissolve NaOH to provide samples with a good color of mycorrhizas for microscopic analysis. Pieces of root 5 cm in size were used for analysis. The MycoPatt methodology [88] was used, which ensures the objective evaluation of the mycorrhizal level in the root cortex by allowing the change of the observation angle using a system of 10 × 10 grids as a microscopic image analysis system.

Thus, colonization maps can be created at different levels of resolution that report to the exact root length. MycoPatt evaluates in detail how mycorrhizae are extended, and each indicator is calculated separately horizontally and vertically for each microscopic field. In this way, the data were obtained objectively and show the real extent of the structures in the roots [89,90].

### 4.6. Soil Sample Collection and Preparation for Analysis for Agrotechnical Purposes

In order to determine the variable properties of the soil, modifiable under the influence of agrotechnical works, it was necessary to collect soil samples. The soil samples were collected as follows: sampling location, 2 different locations in each tunnel; number of samples, 3–4 samples; depth: 10–20 cm; collection times: before transplanting and after the last harvesting). The distribution of the collection points was made according to the configuration of the land by adopting one of the following variants: framing the points uniformly on the entire surface of the analytical unit, on the two diagonals or in a zigzag pattern. In our study we used the last option. A 1–1.5 kg sample of soil was collected for the agrotechnical analyses. The conditioning of the soil samples was performed at OSPA (Office for Pedological and Agrochemical Studies, Cluj-Napoca, Romania). The drying of the soil samples was performed at room temperature, 18–25 °C, for a period of 3–5 days. In the laboratory, the soil samples were protected from sunlight and covered with filter paper to avoid dusting. Until the time of analysis, the soil samples were stored in cardboard boxes or in jars with ground stoppers. The sampling was performed before and after the vegetation cycle period.

### 4.7. Growth Parameters

At the end of the vegetation period, the growth parameters, such as plant height (cm) and the number of leaves per plant, were measured. Harvesting of the tomato fruits started about 50 days after transplanting. The tomato fruits were handpicked every week from the beginning of July to August as they gradually achieved physiological maturity (every 10 days). After each harvest, the fruits were immediately transferred to the laboratory, and the yield parameters were recorded as follows: number of fruits per plant, fresh weight of harvested fruits per plant, fruit height, fruit width, fruit shape index, fruit yield, yield per area, fruit firmness and fruit sugar content.

### 4.8. Determination of Sugar Content (°Brix) and Firmness

Representative samples of uniform unblemished fruits of similar sizes and colors were chosen for the determination of sugar content (°Brix). In order to determine the sugar content of the tomato fruits after each harvest, the fruits were stored for two days at room temperature, after which the measurements were performed. From each genotype, ten fruits were collected from five different harvesting periods. The results were then processed as average values.

For the fruit firmness evaluation, the fruits from five different harvesting periods from each genotype were collected. The fruits were measured at maturity using a Geotester pocket penetrometer T0161 (Giesbeek, Netherlands). The fruit penetrometer measures the degree of penetration and crushing of the fruit pulp using a tip of 1 cm^2^ (7/6″; 11 mm diam) or 1/2 cm^2^ (5116″; 8 mm diam), which offers one of the best possible tests (the pressure is measured in pounds and kg). The accuracy of the penetrometer is + 1% to 2000 g and + 1–2% to 2000 g.

### 4.9. Data Analysis

In order to identify the differences recorded for the soil content in different elements as average values resulting from the three treatments of the experiment, between the beginning of the tomato crop and the end of the crop (cleaning of the culture), the paired *t*-test was applied at the alpha level of 0.05. The registered data of tomato growth, yield and quality parameters were processed as the mean of the traits. An analysis of variance (ANOVA) and a two-way ANOVA (for estimating the interactions between 12 genotypes × three treatments) were applied to the analyzed traits. When the null hypothesis was rejected, a posthoc test was applied to analyze the differences and make multiple comparisons, namely the Duncan Multiple Range Test (Duncan’s MRT, *p* < 0.05). Pearson correlation coefficients were calculated for the traits analyzed (*p* < 0.05), and multivariate analyses were processed using Past software [91].

## 5. Conclusions

Our result suggests that the addition of arbuscular mycorrhiza, without fertilization, can have a beneficial effect on tomato crops. Fertilizers decreased the interaction of tomato roots with beneficial fungi. The biostimulation through the application of AMF seems a promising strategy for improving the results in tomato crops. The plants inoculated with AMF produced more vigorous plants, a better developed root system, bigger fruits, and a higher level of production compared with non-inoculated plants. However, the study highlighted that there are significant differences among genotypes related to their response to mycorrhizae, which shows that not all cultivars react equally favorably to treatments involving mycorrhiza fungi inoculation. The results related to the soil analysis in the main elements indicated different values before planting the tomato seedlings and after the last harvest and removal of plants; arbuscular mycorrhizal fungi probably facilitate the absorption of phosphorus by plants and increase the phosphorus content of the soil. The use of mycorrhiza can be considered as a form of biofertilization that creates favorable symbiosis between plant roots and beneficial organisms in the soil with multiple benefits for plants. This type of agronomic practice may be of interest in agroecosystems where conventional practices are increasingly harmful to the environment.

## Figures and Tables

**Table 1:** Soil content in the main elements before transplanting the tomato seedlings in the three tunnels, where the three treatments (T1, T2 and T3) were applied and after culture (after the clearing of plants at the final harvest).

Elements and Units of Measurement	Before Culture			After Culture		
	Treatment *			Treatment *		
	T1	T2	T3	T1	T2	T3
pH	8.02	7.51	7.70	7.91	7.58	7.84
Humus (%)	1.62	3.88	2.98	2.64	4.31	2.38
Nitrogen—N (%)	0.11	0.25	0.17	0.14	0.26	0.22
Phosphorus—P (ppm)	59	272	211	114	360	264
Potassium—K (ppm)	154	552	264	266	576	376
Calcium—Ca (mg/100 g soil)	6	8	7	9	6.4	6
Magnesium—Mg (mg/100 g soil)	1.82	4.86	4.25	1.21	2.18	3.64
Sodium—Na (mg/100 g soil)	24.5	15.75	21.5	25	17.75	16.75
Potassium—K (mg/100 g soil)	2	11	3	3	9	5
HCO_3_ (mg/100 g soil)	24.4	21.35	21.35	27.45	24.4	18.3
Chlorine—Cl (mg/100 g soil)	39.05	28.4	40.82	40.82	28.4	31.95
SO_4_ (mg/100 g soil)	12.8	44.8	32	32	38.4	32
CaCO_3_ (%)	2.2	3.2	4.8	1.8	1	2.2
Cadmium—Cd (mg/kg)	12.24	13.18	21.75	8.61	12.22	21.24
Copper—Cu (mg/kg)	58.78	71.35	67.13	71.69	66.02	66.9
Nickel—Ni (mg/kg)	43.71	45.84	55.7	77.82	53.62	44.92
Zinc—Zn (mg/kg)	104.24	151.68	120.43	114.9	147.61	111.71
Chromium—Cr (mg/kg)	68.6	57.17	92.5	133.83	73.17	55.05
Lead—Pb (mg/kg)	20.63	43.4	22.65	31.86	24.35	32.66
Manganese—Mn (mg/kg)	889.91	1197.5	942.2	953.1	1201.9	902.9
Cobalt—Co (mg/kg)	4.27	23.02	1.75	8.76	22.69	16.15
Iron—Fe (ppm)	143	206	325	156	168	237
Humidity (%)	16.48	31.02	17.16	16.96	27.76	20.68
Salt content (mS)	0.33	0.3	0.33	0.34	0.3	0.25
N-NO_3_	0.75	2.55	0.15	0.9	1.05	1.35
N-NH_4_	0.84	0.63	0.21	0.84	0.42	0.42

* T1: control, without fertilizer and mycorrhizae colonization; T2: NPK and microelements fertigation without mycorrhizae colonization; and T3: arbuscular mycorrhizal fungi (AMF), seedling roots inoculated with specialized soil-borne fungi.

**Table 2:** Changes in the soil components between the two periods: A, after culture (after the clearing of plants at the final harvest and end of crop); and B, before culture (at the planting seedlings and start of the culture). The results are the average values ± SD (standard deviation) of the three treatments (T1, T2, T3).

Elements and Units of Measurement.	Before Culture (B)	After Culture (A)	Difference (A-B) (±)	Significance (‘*p*’ Value)
pH	7.74 ± 0.26	7.78 ± 0.17	0.04	0.698
Humus (%)	2.83 ± 1.14	3.11 ± 1.05	0.28	0.610
Nitrogen—N (%)	0.18 ± 0.07	0.21 ± 0.06	0.03	0.122
Phosphorus—P (ppm)	180.67 ± 109.69	246.00 ± 123.98	65.33	0.029 *
Potassium—K (ppm)	323.33 ± 205.53	406.00 ± 157.16	82.67	0.106
Calcium—Ca (mg/100 g soil)	7.00 ± 1.00	7.13 ± 1.63	0.13	0.935
Magnesium—Mg (mg/100 g soil)	3.64 ± 1.61	2.34 ± 1.22	−1.30	0.200
Sodium—Na (mg/100 g soil)	20.58 ± 4.45	19.83 ± 4.50	−0.75	0.749
Potassium—K (mg/100 g soil)	5.33 ± 4.93	5.67 ± 3.06	0.33	0.808
HCO_3_ (mg/100 g soil)	22.37 ± 1.76	23.38 ± 4.66	1.02	0.667
Chlorine—Cl (mg/100 g soil)	36.09 ± 6.72	33.72 ± 6.40	−2.37	0.547
SO_4_ (mg/100 g soil)	29.87 ± 16.11	34.13 ± 3.70	4.27	0.635
CaCO_3_ (%)	3.40 ± 1.31	1.67 ± 0.61	−1.73	0.125
Cadmium—Cd (mg/kg)	15.72 ± 5.24	14.02 ± 6.51	−1.70	0.223
Copper—Cu (mg/kg)	65.75 ± 6.40	68.20 ± 3.05	2.45	0.696
Nickel—Ni (mg/kg)	48.42 ± 6.40	58.79 ± 17.05	10.37	0.509
Zinc—Zn (mg/kg)	125.45 ± 24.12	124.74 ± 19.87	−0.71	0.914
Chromium—Cr (mg/kg)	72.76 ± 18.03	87.35 ± 41.26	14.59	0.671
Lead—Pb (mg/kg)	28.89 ± 12.60	29.62 ± 4.58	0.73	0.948
Manganese—Mn (mg/kg)	1009.86 ± 164.57	1019.31 ± 160.13	9.45	0.780
Cobalt—Co (mg/kg)	9.68 ± 11.62	15.87 ± 6.97	6.19	0.290
Iron—Fe (ppm)	224.67 ± 92.42	187.00 ± 43.71	−37.67	0.326
Humidity (%)	21.55 ± 8.21	21.80 ± 5.49	0.25	0.911
Salt content (mS)	0.32 ± 0.02	0.30 ± 0.05	−0.02	0.499
N–NO_3_	1.15 ± 1.25	1.10 ± 0.23	−0.05	0.955
N–NH_4_	0.56 ± 0.32	0.56 ± 0.24	0.00	0.999

* The asterisk indicates significant differences for α < 0.05 (paired *t*-test).

**Table 3:** The main characteristics of biomass of tomato plants according to genotype and treatment.

Genotypes (G)	Length of Roots (cm)			Mean ± SD (G)	Plant Height (cm)			Mean ± SD (G)	Number of Leaves/Plant			Mean ± SD (G)
	T1	T2	T3		T1	T2	T3		T1	T2	T3	
Precos	16.5 ^ab^	16.4 ^ab^	17.4 ^a–c^	16.8 ± 0.6 ^A^	114.0 ^d^	122.0 ^f–i^	129.5 ^j–m^	121.8 ± 7.8 ^BC^	8.0 ^b–d^	9.0 ^c–e^	10.0 ^e–g^	9.0 ± 1.0 ^B^
AS28♀F6	24.4 ^g–k^	24.6 ^g–k^	26.7 ^k–m^	25.2 ± 1.3 ^CD^	114.0 ^d^	121.0 ^e–h^	127.0 ^i–l^	120.7 ± 6.5 ^BC^	9.0 ^c–e^	10.5 ^e–h^	11.0 ^f–i^	10.2 ± 1.0 ^CD^
AS29♂F6	15.8 ^ab^	15.7 ^ab^	18.3 ^b–d^	16.6 ± 1.5 ^A^	56.0 ^a^	56.0 ^a^	60.5 ^ab^	57.5 ± 2.6 ^A^	6.0 ^a^	6.5 ^ab^	6.5 ^ab^	6.5 ± 0.3 ^A^
AS30♀F7	25.6 ^i–l^	27.5 ^lm^	29.2 ^m^	27.4 ± 1.8 ^D^	120.0 ^e–h^	123.5 ^g–i^	129.5 ^j–m^	124.3 ± 4.8 ^CD^	9.0 ^c–e^	9.5 ^d–f^	11.0 ^f–i^	9.8 ± 1.0 ^BC^
AS31♂F7	15.1 ^a^	15.9 ^ab^	19.3 ^cd^	16.8 ± 2.2 ^A^	57.5 ^a^	59.5 ^ab^	63.5 ^b^	60.2 ± 3.1 ^A^	6.0 ^a^	6.0 ^a^	7.5 ^a–c^	6.5 ± 0.9 ^A^
AS300	22.5 ^f–h^	24.0 ^g–j^	25.1 ^h–l^	23.9 ± 1.3 ^C^	115.5 ^de^	132.0 ^lm^	144.0 ^no^	130.5 ± 14.3 ^DE^	10.5 ^e–h^	12.0 ^h–j^	12.5 ^i–j^	16.7 ± 1.0 ^EF^
Addalyn	23.3 ^g–i^	23.2 ^g–i^	24.8 ^g–k^	23.8 ± 0.9 ^C^	124.0 ^g–j^	134.5 ^m^	143.5 ^no^	134.0 ± 9.8 ^EF^	10.5 ^e–h^	10.0 ^e–g^	12.0 ^h–j^	10.8 ± 1.0 ^DE^
AS07♀F6	24.3 ^g–k^	25.5 ^i–l^	27.7 ^lm^	25.8 ± 1.7 ^CD^	125.5 ^h–k^	133.0 ^m^	140.5 ^n^	133.0 ± 7.5 ^EF^	9.5 ^d–f^	10.5 ^e–h^	10.0 ^e–g^	10.0 ± 0.5 ^CD^
AS08♂F6	17.5 ^a–c^	17.8 ^bc^	20.7 ^d–f^	18.7 ± 1.8 ^AB^	107.0 ^c^	117.0 ^d–f^	124.5 ^g–k^	116.2 ± 8.8 ^B^	9.0 ^c–e^	9.0 ^c–e^	9.0 ^c–e^	9.0 ± 0.0 ^B^
AS09♀F7	25.2 ^i–l^	26.2 ^j–l^	28.9 ^m^	26.8 ± 1.9 ^D^	125.0 ^g–k^	140.0 ^n^	149.0 ^o^	138.0 ± 12.1 ^F^	9.5 ^d–f^	10.5 ^e–h^	12.5 ^i–j^	10.8 ± 1.5 ^DE^
AS10♂F7	18.1 ^bc^	19.8 ^c–e^	22.2 ^e–g^	20.0 ± 2.1 ^B^	119.0 ^d–g^	119.0 ^d–g^	130.0 ^k–m^	122.7 ± 6.4 ^BC^	9.5 ^d–f^	9.5 ^d–f^	9.5 ^d–f^	9.5 ± 0.0 ^BC^
AS400	23.9 ^g–j^	25.8 ^i–l^	27.5 ^lm^	25.8 ± 1.8^C D^	130.0 ^k–m^	140.0 ^n^	148.0 ^o^	139.3 ± 9.0 ^F^	11.0 ^f–i^	11.5 ^g–j^	13.0 ^j^	11.8 ± 1.0 ^F^
Mean ± SD (T)	21.0 ± 4.0 ^X^	21.9 ± 4.4 ^Y^	24.0 ± 4.2 ^Z^	-	108.9 ± 25.2 ^X^	116.5 ± 28.5 ^Y^	124.1 ± 30.2 ^Z^	-	8.9 ± 1.6 ^X^	9.5 ± 1.8 ^Y^	10.4 ± 2.0 ^Z^	-

All values are expressed as means. For genotype (G) and treatment (T) influence, mean ± standard deviation (SD) is shown. Within each trait and each column represented by the treatments T1, T2 and T3, any two means followed by the same lower case are not significantly different; the respective mean values reflect the interaction between genotype and treatment. The mean values in the columns of each trait (average of T1, T2, and T3) reflect the unilateral effect of the genotype, regardless of the other experimental factor (treatment). Any two means followed by the same uppercase letters are not significantly different. The average values in the last row reflect the unilateral effect of the treatment, regardless of the other experimental factor (genotype). Any two means within each trait followed by the same uppercase letters are not significantly different. Duncan’s multiple range test was used as a posthoc test (DMRT Duncan test, α < 0.05).

**Table 4:** Dry root weight and dry shoots weight of tomato plants according to genotype and treatment.

Genotypes (G)	Dry Root Weight (g)			Mean ± SD (G)	Dry Shoot Weight (g)			Mean ± SD (G)
	T1	T2	T3		T1	T2	T3	
Precos	0.66 ^c^	0.66 ^c^	0.81 ^cd^	0.71 ± 0.09 ^B^	10.5 ^a^	10.7 ^a^	11.2 ^ab^	10.8 ± 0.4 ^A^
AS28♀F6	1.29 ^e^	1.43 ^e–g^	1.32 ^ef^	1.35 ± 0.07 ^C^	16.2 ^ef^	17.2 ^f–h^	17.8 ^g–i^	17.1 ± 0.8 ^B^
AS29♂F6	0.37 ^ab^	0.41 ^b^	0.38 ^ab^	0.39 ± 0.02 ^A^	12.4 ^b^	11.1 ^ab^	11.9 ^ab^	11.8 ± 0.7 ^A^
AS30♀F7	0.87 ^d^	0.71 ^c^	0.78 ^cd^	0.79 ± 0.08 ^B^	14.7 ^cd^	16.1 ^d–f^	16.1 ^d–f^	15.7 ± 0.8 ^B^
AS31♂F7	0.26 ^a^	0.38 ^ab^	0.32 ^ab^	0.32 ± 0.06 ^A^	10.7 ^a^	11.7 ^ab^	12.2 ^b^	11.7 ± 0.8 ^A^
AS300	1.70 ^j–l^	1.83 ^lm^	1.90 ^mn^	1.81 ± 0.10 ^F^	19.0 ^i–l^	20.6 ^m^	20.9 ^m^	20.2 ± 1.0 ^C^
Addalyn	1.59 ^h–j^	1.70 ^j–l^	1.71 ^j–l^	1.67 ± 0.07 ^E^	18.1 ^h–j^	19.9 ^k–m^	19.0 ^i–l^	19.0 ± 0.9 ^C^
AS07♀F6	1.44 ^f–h^	1.48 ^g–i^	1.60 ^ij^	1.51 ± 0.08 ^D^	14.5 ^c^	15.5 ^c–e^	16.3 ^ef^	15.4 ± 0.9 ^B^
AS08♂F6	1.56 ^g–j^	1.68 ^jk^	1.46 ^f–i^	1.57 ± 0.11 ^DE^	16.1 ^d–f^	16.6 ^e–g^	16.0 ^d–f^	16.2 ± 0.3 ^B^
AS09♀F7	2.00 ^no^	2.07 ^o^	2.23 ^p^	2.10 ± 0.12 ^G^	20.8 ^m^	18.6 ^i–k^	20.9 ^m^	20.1 ± 1.3 ^C^
AS10♂F7	1.80 ^k–m^	1.90 ^mn^	2.04 ^no^	1.91 ± 0.12 ^F^	19.1 ^i–l^	19.8 ^k–m^	19.1 ^i–l^	19.4 ± 0.4 ^C^
AS400	2.06 ^o^	2.07 ^o^	2.37 ^q^	2.17 ± 0.18 ^G^	19.2 ^j–l^	20.1 ^lm^	21.1 ^m^	20.2 ± 1.0 ^C^
Mean ± SD (T)	1.30 ± 0.62 ^X^	1.36 ± 0.64 ^Y^	1.41 ± 0.70 ^Z^	-	15.97 ± 3.5 ^X^	16.53 ± 3.6 ^Y^	16.90 ± 3.6 ^Z^	-

The explanations regarding the treatments (T1, T2, and T3) and the significance of the genotype × treatment interaction, the effect of the genotype and the effect of the treatment are given in the caption of Table 3. Duncan’s multiple range test was used as a posthoc test (DMRT Duncan test, α < 0.05).

**Table 5:** The main characteristics of fruits according to genotype and treatment.

Genotypes (G)	Fruit Height (cm)			Mean ± SD (G)	Fruit Width (cm)			Mean ± SD (G)	Fruit Shape Index			Mean ± SD (G)	Fruit Weight (g)			Mean ± SD (G)
	T1	T2	T3		T1	T2	T3		T1	T2	T3		T1	T2	T3	
Precos	6.5 ^ab^	6.0 ^a^	6.5 ^ab^	6.3 ± 0.3 ^A^	6.5 ^a^	6.5 ^a^	7.0 ^ab^	6.7 ± 0.3 ^A^	1.00 ^k^	0.93 ^i–k^	0.93 ^i–k^	0.95 ± 0.04 ^D^	100.0 ^a^	92.5 ^a^	112.0 ^b^	101.5 ± 9.8 ^A^
AS28♀F6	7.7 ^c–f^	8.2 ^e–g^	7.0 ^a–d^	7.6 ± 0.6 ^BC^	9.2 ^e^	8.5 ^c–e^	8.0 ^b–d^	8.6 ± 0.6 ^B^	0.83 ^d–j^	0.97 ^jk^	0.88 ^f–k^	0.89 ± 0.07 ^CD^	154.5 ^c^	156.0 ^c^	160.5 ^c^	157.0 ± 3.1 ^B^
AS29♂F6	8.0 ^d–g^	7.7 ^c–f^	7.5 ^b–f^	7.7 ± 0.3 ^C^	11.2 ^gh^	11.0 ^gh^	11.0 ^gh^	11.1 ± 0.1 ^C^	0.71 ^a–e^	0.70 ^a–e^	0.68 ^a–c^	0.70 ± 0.02 ^A^	237.0 ^j–l^	240.0 ^kl^	248.5 ^l^	241.8 ± 6.0 ^F^
AS30♀F7	8.0 ^d–g^	6.7 ^a–c^	6.7 ^a–c^	7.1 ± 0.8 ^B^	9.5 ^ef^	7.5 ^a–c^	8.0 ^b–d^	8.3 ± 1.0 ^B^	0.84 ^e–j^	0.90 ^g–k^	0.84 ^e–j^	0.86 ± 0.03 ^CD^	154.5 ^c^	160.0 ^c^	164.5 ^c^	159.7 ± 5.0 ^B^
AS31♂F7	8.5 ^fg^	8.0 ^d–g^	8.5 ^fg^	8.3 ± 0.3 ^D^	11.2 ^gh^	11.0 ^gh^	11.5 ^g–i^	11.2 ± 0.3 ^CD^	0.76 ^b–g^	0.73 ^a–e^	0.74 ^a–f^	0.74 ± 0.02 ^AB^	225.0 ^h–j^	230.5 ^i–k^	238.5 ^j–l^	231.3 ± 6.8 ^EF^
AS300	8.2 ^e–g^	7.7 ^c–f^	8.0 ^d–g^	8.0±0.3^CD^	10.5 ^fg^	11.7 ^hi^	11.5 ^g–i^	11.2 ± 0.6 ^CD^	0.78 ^b–h^	0.66 ^ab^	0.69 ^a–d^	0.71 ± 0.06 ^A^	199.0 ^de^	192.0 ^d^	214.5 ^f–h^	201.8 ± 11.5 ^C^
Addalyn	7.0 ^a–d^	7.2 ^b–e^	7.2 ^b–e^	7.1 ± 0.1 ^B^	7.2 ^ab^	9.2 ^e^	9.5 ^ef^	8.6 ± 1.3 ^B^	0.96 ^jk^	0.78 ^b–h^	0.76 ^b–g^	0.83 ± 0.11 ^BC^	191.0 ^d^	198.0 ^de^	228.5 ^i–k^	205.8 ± 19.9 ^C^
AS07♀ F6	7.7 ^c–f^	7.0 ^a–d^	6.7 ^a–c^	7.1 ± 0.5 ^B^	9.2 ^e^	7.5 ^a–c^	7.5 ^a–c^	8.1 ± 1.0 ^B^	0.84 ^e–j^	0.94 ^i–k^	0.90 ^g–k^	0.89 ± 0.05 ^CD^	161.5 ^c^	158.5 ^c^	160.5 ^c^	160.2 ± 1.5 ^B^
AS08♂F6	9.0 ^g^	8.5^fg^	7.7 ^c–f^	8.4 ± 0.7 ^D^	11.0 ^gh^	12.0 ^hi^	11.5 ^g–i^	11.5 ± 0.5 ^CD^	0.82 ^c–i^	0.71 ^a–e^	0.67 ^ab^	0.73 ± 0.08 ^A^	225.0 ^h–j^	221.0 ^g–i^	230.5 ^i–k^	225.5 ± 4.8 ^E^
AS09♀F7	8.2^e–g^	7.5 ^b–f^	7.0 ^a–d^	7.6 ± 0.6 ^BC^	9.0 ^de^	7.5 ^a–c^	7.5 ^a–c^	8.0 ± 0.9 ^B^	0.91 ^h–k^	1.00 ^k^	0.94^i–k^	0.95 ± 0.05 ^D^	163.0 ^c^	153.0 ^c^	156.0 ^c^	157.3 ± 5.1 ^B^
AS10♂F7	8.2 ^e–g^	7.7 ^c–f^	8.2 ^e–g^	8.0 ± 0.3 ^CD^	11.0 ^gh^	11.2 ^gh^	11.0 ^gh^	11.1 ± 0.1 ^C^	0.75 ^a–f^	0.69 ^a–c^	0.75 ^a–f^	0.73 ± 0.03 ^A^	212.5 ^f–h^	222.5 ^g–i^	229.0 ^i–k^	221.3 ± 8.3 ^DE^
AS400	8.2 ^e–g^	7.7 ^c–f^	7.7 ^c–f^	7.9 ± 0.3 ^CD^	11.2 ^gh^	11.7 ^hi^	12.5 ^i^	11.8 ± 0.7 ^D^	0.73 ^a–f^	0.66 ^ab^	0.62 ^a^	0.67 ± 0.06 ^A^	203.5 ^d–f^	209.5 ^e–g^	220.5 ^g–i^	211.2 ± 8.6 ^CD^
Mean ± SD (T)	7.9 ± 0.7 ^X^	7.5 ± 0.7 ^Y^	7.4 ± 0.6 ^Z^	-	9.7 ± 1.6 ^X^	9.6 ± 2.0 ^Y^	9.7 ± 2.0 ^Z^	-	0.83 ± 0.09 ^X^	0.81 ± 0.13 ^Y^	0.78 ± 0.11 ^Z^	-	185.5 ± 39.7 ^X^	186.1 ± 43.0 ^Y^	197.0 ± 43.7 ^Z^	-

The explanations regarding the treatments (T1, T2, and T3) and the significance of the genotype *×* treatment interaction, the effect of the genotype and the effect of the treatment are given in the caption of Table 3. Duncan’s multiple range test was used as a posthoc test (DMRT Duncan test, α < 0.05).

**Table 6:** The main quantitative parameters of tomato production according to genotype and treatment.

Genotypes (G)	Number of Fruit/Plants			Mean ± SD (G)	Yield/Plant (kg)			Mean ± SD (G)	Yield/Area (kg)			Mean ± SD (G)
	T1	T2	T3		T1	T2	T3		T1	T2	T3	
Precos	11.5 ^a–c^	12.0 ^a–d^	10.5 ^ab^	11.3 ± 0.8 ^AB^	1.25 ^a^	1.35 ^a^	1.20 ^a^	1.3 ± 0.1 ^A^	4.13 ^a^	4.45 ^a^	3.96 ^a^	4.18 ± 0.25 ^A^
AS28♀F6	16.0 ^e–i^	14.5 ^c–f^	15.0 ^d–g^	15.2 ± 0.8 ^C^	2.35 ^b–d^	2.05 ^b^	2.20 ^bc^	2.2 ± 0.2 ^B^	7.76 ^b–d^	6.77 ^b^	7.26 ^bc^	7.26±0.50 ^B^
AS29♂F6	9.5 ^a^	10.5 ^ab^	11.5 ^a–c^	10.5 ± 1.0 ^AB^	2.95 ^d–g^	2.70 ^b–f^	3.30 ^fg^	3.0 ± 0.3 ^D^	9.73 ^d–g^	8.91 ^b–f^	10.89 ^fg^	9.84 ± 0.99 ^D^
AS30♀F7	18.0 ^g–i^	16.5 ^f–i^	14.5 ^c–f^	16.3 ± 1.8 ^CD^	2.60 ^b–f^	2.45 ^b–d^	2.35 ^b–d^	2.5 ± 0.1 ^BC^	8.58 ^b–f^	8.09 ^b–d^	7.76 ^b–d^	8.14 ± 0.41 ^BC^
AS31♂F7	10.0 ^ab^	10.0^ab^	10.0 ^ab^	10.0 ± 0.0 ^A^	2.40 ^b–d^	2.20 ^bc^	2.70 ^b–f^	2.4 ± 0.3 ^BC^	7.92 ^b–d^	7.26 ^bc^	8.91 ^b–f^	8.03 ± 0.83 ^BC^
AS300	16.0 ^e–i^	15.5 ^e–h^	15.5 ^e–h^	15.7 ± 0.3 ^C^	2.95 ^d–g^	2.80 ^c–f^	2.95 ^d–g^	2.9 ± 0.1 ^D^	9.73 ^d–g^	9.24 ^c–f^	9.73 ^d–g^	9.57 ± 0.28 ^D^
Addalyn	17.0 ^f–i^	17.5 ^f–i^	18.0 ^g–i^	17.5 ± 0.5 ^DE^	3.25 ^e–g^	3.25 ^e–g^	3.65 ^gh^	3.4 ± 0.2 ^E^	10.73 ^e–g^	10.73 ^e–g^	12.05 ^gh^	11.17 ± 0.76 ^E^
AS07♀F6	17.0 ^f–i^	17.0 ^f–i^	16.0 ^e–i^	16.7 ± 0.6 ^CD^	2.55 ^b–e^	2.95 ^d–g^	2.25 ^b–d^	2.6 ± 0.4 ^C^	8.41 ^b–e^	9.73 ^d–g^	7.43 ^b–d^	8.52 ± 1.15 ^C^
AS08♂F6	12.0 ^a–d^	13.0 ^b–e^	11.0 ^ab^	12.0 ± 1.0 ^B^	2.25 ^b–d^	2.85 ^c–f^	2.60 ^b–f^	2.6 ± 0.3 ^C^	7.43 ^b–d^	9.41 ^c–f^	8.58 ^b–f^	8.47 ± 0.99 ^C^
AS09♀F7	16.5 ^f–i^	16.5 ^f–i^	15.0 ^d–g^	16.0 ± 0.9 ^CD^	2.20 ^bc^	2.70 ^b–f^	2.30 ^b–d^	2.4 ± 0.3 ^BC^	7.26 ^bc^	8.91 ^b–f^	7.59 ^b–d^	7.92 ± 0.87 ^BC^
AS10♂F7	12.0 ^a–d^	11.0 ^ab^	12.0 ^a–d^	11.7 ± 0.6 ^AB^	2.25 ^b–d^	2.20 ^bc^	2.40 ^b–d^	2.3 ± 0.1 ^BC^	7.43 ^b–d^	7.26 ^bc^	7.92 ^b–d^	7.54 ± 0.34 ^BC^
AS400	19.0 ^i^	19.0 ^i^	18.5 ^hi^	18.8 ± 0.3 ^E^	4.05 ^hi^	4.35 ^i^	4.15 ^hi^	4.2 ± 0.2 ^F^	13.32 ^hi^	14.35 ^i^	13.69 ^hi^	13.79 ± 0.52 ^F^
Mean ± SD (T)	14.5 ± 3.3 ^X^	14.4 ± 3.0 ^Y^	13.9 ± 2.9 ^Z^	-	2.59 ± 0.68 ^X^	2.65 ± 0.73 ^Y^	2.67 ± 0.77 ^Z^	-	8.54 ± 2.24 ^X^	8.76 ± 2.42 ^Y^	8.81 ± 2.54 ^Z^	

The explanations regarding the treatments (T1, T2, and T3) and the significance of the genotype × treatment interaction, the effect of the genotype and the effect of the treatment are given in the caption of Table 3. Duncan’s multiple range test was used as a posthoc test (DMRT Duncan test, α < 0.05).

**Table 7:** Fruit firmness and sugar content of the fruits according to genotype and treatment.

Genotypes (G)	Fruit Firmness (kg/cm^2^)			Mean ± SD (G)	Sugar Content (°Brix)			Mean ± SD (G)
	T1	T2	T3		T1	T2	T3	
Precos	4.5 ^a–c^	4.0 ^a^	4.7 ^b–d^	4.4 ± 0.4 ^A^	4.6 ^ab^	4.6 ^ab^	5.0 ^c–g^	4.7 ± 0.2 ^A–C^
AS28♀F6	5.7 ^f–h^	6.0 ^g–i^	6.5 ^i–k^	6.1 ± 0.4 ^CD^	4.6 ^ab^	4.6 ^ab^	4.7 ^ab^	4.6 ± 0.1 ^AB^
AS29♂F6	4.7 ^b–d^	4.7 ^b–d^	5.0 ^c–e^	4.8 ± 0.2 ^A^	4.8 ^a–d^	4.7 ^ab^	5.2 ^f–h^	4.9 ± 0.3 ^B–D^
AS30♀F7	7.0 ^k^	5.7 ^f–h^	6.2 ^h–j^	6.3 ± 0.7 ^CD^	4.6 ^ab^	4.6 ^ab^	4.7 ^a–c^	4.6 ± 0.1 ^A–C^
AS31♂F7	5.0 ^c–e^	4.7 ^b–d^	5.2 ^d–f^	5.0 ± 0.3 ^AB^	4.6 ^ab^	4.5 ^a^	4.8 ^a–c^	4.6 ± 0.2 ^A–C^
AS300	6.0 ^g–i^	6.0 ^g–i^	6.5 ^i–k^	6.2 ± 0.3 ^CD^	5.2 ^f–h^	5.3 ^gh^	5.4 ^h^	5.3 ± 0.1 ^E^
Addalyn	5.5 ^e–g^	5.5 ^e–g^	6.0 ^g–i^	5.7 ± 0.3 ^BC^	4.6 ^ab^	4.7 ^a–c^	4.7 ^a–c^	4.7 ± 0.1 ^A–C^
AS07♀F6	6.2 ^h–j^	6.0 ^g–i^	6.2 ^h–j^	6.1 ± 0.1 ^CD^	4.5 ^a^	4.6 ^ab^	4.7 ^ab^	4.6 ± 0.1 ^AB^
AS08♂F6	5.0 ^c–e^	4.2 ^ab^	4.7 ^b–d^	4.6 ± 0.4 ^A^	5.3 ^gh^	4.8 ^a–c^	5.1 ^d–h^	5.1 ± 0.3 ^DE^
AS09♀F7	6.5 ^i–k^	6.2 ^h–j^	7.0 ^k^	6.6 ± 0.4 ^D^	4.6 ^ab^	4.6 ^ab^	4.5 ^a^	4.6 ± 0.1 ^A^
AS10♂F7	5.2 ^d–f^	4.7 ^b–d^	5.0 ^c–e^	5.0 ± 0.3 ^AB^	5.1 ^d–h^	5.0 ^c–g^	5.2 ^e–h^	5.1 ± 0.1 ^DE^
AS400	6.7 ^jk^	6.0 ^g–i^	6.0 ^g–i^	6.2 ± 0.4 ^CD^	4.9 ^a–e^	4.9 ^b–f^	5.0 ^c–g^	4.9 ± 0.1 ^CD^
Mean ± SD (T)	5.7 ± 0.8 ^X^	5.3 ± 0.8 ^Y^	5.8 ± 0.8 ^Z^	-	4.8 ± 0.3 ^X^	4.7 ± 0.2 ^Y^	4.9 ± 0.3 ^Z^	-

The explanations regarding the treatments (T1, T2, and T3) and the significance of the genotype × treatment interaction, the effect of the genotype and the effect of the treatment are given in the caption of Table 3. Duncan’s multiple range test was used as a posthoc test (DMRT Duncan test, α < 0.05).

**Table 8:** The combined influence of genotype and treatment on mycorrhizal colonization.

Genotypes/ Treatment (G/T)		Frequency (%)	Intensity (%)	Non-Mycorrhizal Areas (%)	Degree of Colonization %	Ratio of Mycorrhizal Areas/Non-Mycorrhizal Areas
Precos	T1	26.0 ± 14.2 ^a–e^	9.3 ± 2.5 ^b–h^	90.6 ± 2.5 ^e–k^	2.6 ± 2.1 ^a–e^	0.11 ± 0.03 ^a–g^
	T2	10.7 ± 4.6 ^a–c^	5.0 ± 0.6 ^a–b^	95.6 ± 0.6 ^k–l^	0.4 ± 0.3 ^a^	0.04 ± 0.01 ^ab^
	T3	32.7 ± 11.4 ^c–h^	7.0 ± 4.4 ^a–e^	92.0 ± 4.4 ^g–l^	2.8 ± 2.3 ^a–f^	0.09 ± 0.05 ^a–f^
AS28♀F6	T1	13.0 ± 1.7 ^a–c^	16.6 ± 1.0 ^jk^	95.0 ± 1.0 ^j–l^	0.6 ± 0.1 ^ab^	0.05 ± 0.01 ^a–c^
	T2	14.0 ± 5.3 ^a–c^	12.6 ± 1.0 ^e–k^	93.0 ± 1.0 ^h–l^	0.9 ± 0.4 ^ab^	0.08 ± 0.01 ^a–e^
	T3	43.7 ± 21.8 ^d–i^	15.0 ± 3.2 ^h–k^	83.3 ± 3.2 ^bc^	7.7 ± 4.7 ^g–i^	0.20 ± 0.05 ^i–k^
AS29♂F6	T1	41.3 ± 7.1 ^d–i^	16.6 ± 3.1 ^jk^	87.3 ± 3.1 ^b–h^	5.3 ± 2.2 ^b–i^	0.15 ± 0.04 ^e–k^
	T2	41.7 ± 18.9 ^d–i^	13.3 ± 3.0 ^f–k^	85.0 ± 3.0 ^b–e^	8.3 ± 1.8 ^hi^	0.18 ± 0.04 ^g–k^
	T3	46.7 ± 17.6 ^e–i^	16.6 ± 5.5 ^jk^	83.3 ± 5.5 ^bc^	8.3 ± 5.6 ^hi^	0.20 ± 0.08 ^jk^
AS30♀F7	T1	54.7 ± 6.1 ^hi^	13.3 ± 1.5 ^f–k^	86.6 ± 1.5 ^b–g^	7.3 ± 1.5 ^e–i^	0.16 ± 0.02 ^f–k^
	T2	44.3 ± 17.8 ^d–i^	14.0 ± 4.0 ^g–k^	86.0 ± 4.0 ^b–f^	6.6 ± 4.2 ^c–i^	0.16 ± 0.04 ^f–k^
	T3	80.3 ± 0.6 ^j^	24.6 ± 5.9 ^l^	75.3 ± 5.9 ^a^	19.8 ± 4.7 ^j^	0.33 ± 0.10 ^l^
AS31♂F7	T1	24.3 ± 8.0 ^a–d^	8.0 ± 2.0 ^a–f^	92.0 ± 2.0 ^g–l^	2.0 ± 1.1 ^a–c^	0.09 ± 0.02 ^a–f^
	T2	13.3 ± 9.5 ^a–c^	7.0 ± 2.6 ^a–e^	93.0 ± 2.6 ^h–l^	1.1 ± 0.5 ^ab^	0.07 ± 0.03 ^a–e^
	T3	13.3 ± 9.5 ^a–c^	5.0 ± 1.0 ^a–c^	95.0 ± 1.0 ^j–l^	0.5 ± 0.3 ^ab^	0.05 ± 0.01 ^a–c^
AS300	T1	25.3 ± 8.1 ^a–e^	6.0 ± 1.0 ^a–d^	94.0 ± 1.0 ^i–l^	1.6 ± 0.7 ^ab^	0.06 ± 0.01 ^a–d^
	T2	30.7 ± 9.2 ^c–g^	11.0 ± 2.6 ^d–i^	89.0 ± 2.6 ^d–i^	3.5 ± 1.7 ^a^	0.13 ± 0.03 ^c–j^
	T3	6.0 ± 5.3 ^a^	2.6 ± 1.2 ^a^	97.3 ± 1.2 ^l^	0.2 ± 0.1 ^a–g^	0.03 ± 0.01 ^a^
Addalyn	T1	60.3 ± 13.9 ^i^	12.0 ± 2.6 ^e–j^	88.0 ± 2.6 ^c–h^	7.4 ± 3.2 ^f–i^	0.14 ± 0.03 ^d–j^
	T2	14.0 ± 6.9 ^a–c^	5.3 ± 1.2 ^a–d^	94.6 ± 1.2 ^i–l^	0.8 ± 0.2 ^ab^	0.05 ± 0.01 ^a–c^
	T3	23.0 ± 8.7 ^a–d^	5.3 ± 4.2 ^a–d^	95.6 ± 4.2 ^i–l^	2.0 ± 0.5 ^a–c^	0.06 ± 0.05 ^a–d^
AS07♀F6	T1	42.0 ± 15.9 ^d–i^	16.3 ± 2.1 ^i–k^	83.6 ± 2.1 ^b–d^	6.9 ± 3.3 ^d–i^	0.19 ± 0.03 ^h–k^
	T2	8.0 ± 6.9 ^ab^	3.0 ± 1.0 ^a^	97.0 ± 1.0 ^l^	0.2 ± 0.1 ^a^	0.03 ± 0.01 ^a^
	T3	44.6 ± 14.2 ^d–i^	10.3 ± 2.5 ^c–h^	89.6 ± 2.5 ^e–j^	4.8 ± 2.7 ^a–i^	0.12 ± 0.03 ^b–h^
AS08♂F6	T1	48.6 ± 8.1 ^f–i^	13.3 ± 4.9 ^f–k^	86.6 ± 4.9 ^b–g^	6.5 ± 2.7 ^c–i^	0.15 ± 0.07 ^e–k^
	T2	23.8 ± 1.5 ^a–d^	9.0 ± 2.0 ^b–g^	92.0 ± 2.0 ^f–k^	2.2 ± 1.5 ^a–d^	0.10 ± 0.02 ^a–g^
	T3	52.7 ± 12.1 ^g–i^	17.6 ± 2.5 ^k^	82.3 ± 2.5 ^b^	9.4 ± 3.0 ^i^	0.22 ± 0.04 ^k^
AS09♀F7	T1	42.0 ± 7.2 ^d–i^	9.6 ± 1.5 ^b–h^	90.3 ± 1.5 ^e–k^	4.3 ± 1.3 ^a–h^	0.11 ± 0.02 ^a–g^
	T2	12.3 ± 4.9 ^a–c^	4.6 ± 1.5 ^a–c^	95.3 ± 1.5 ^j–l^	0.6 ± 0.4 ^ab^	0.05 ± 0.02 ^a–c^
	T3	12.6 ± 10.0 ^a–c^	5.0 ± 2.0 ^a–c^	95.0 ± 2.0 ^j–l^	0.7 ± 0.2 ^ab^	0.05 ± 0.02 ^a–c^
AS10♂F7	T1	39.3 ± 9.0 ^d–i^	10.0 ± 2.0 ^b–h^	90.0 ± 2.0 ^e–l^	4.0 ± 1.7 ^a–h^	0.11 ± 0.02 ^b–g^
	T2	29.0 ± 17.3 ^b–f^	12.0 ± 3.6 ^e–j^	88.0 ± 3.6 ^c–h^	3.4 ± 1.9 ^a–g^	0.14 ± 0.05 ^d–j^
	T3	17.3 ± 6.4 ^a–c^	7.3 ± 2.1 ^a–e^	92.6 ± 2.1 ^h–l^	1.3 ± 0.7 ^ab^	0.08 ± 0.02 ^a–e^
AS400	T1	15.0 ± 5.0 ^a–c^	5.0 ± 0.0 ^a–c^	95.0 ± 0.1 ^j–l^	0.7 ± 0.3 ^ab^	0.05 ± 0.00 ^a–c^
	T2	39.0 ± 5.2 ^d–i^	11.0 ± 2.0 ^d–i^	89.0 ± 2.0 ^d–i^	4.2 ± 0.9 ^a–h^	0.12 ± 0.03 ^b–i^
	T3	15.3 ± 3.1 ^a–c^	9.0 ± 2.0 ^b–g^	91.0 ± 2.0 ^f–k^	1.4 ± 0.5 ^ab^	0.10 ± 0.02 ^a–g^

Any two means on each column followed by the same letters are not significantly different. Duncan’s multiple range test was used as a posthoc test (DMRT Duncan test, α < 0.05).

## Data Availability

Not applicable.
